# Dauciform roots affect functional traits of *Carex filispica* under nitrogen and phosphorus fertilization in alpine meadow

**DOI:** 10.1038/s41598-023-40828-9

**Published:** 2023-08-30

**Authors:** Rong Fan, Jinguo Hua, Songlin Jiang, Xiaoqi Wang, Wanting Liu, Wenli Ji

**Affiliations:** College of Landscape Architecture and Arts, Northwest A&F University, Yangling, 712100 Shaanxi China

**Keywords:** Plant sciences, Environmental sciences

## Abstract

Over recent decades, there has been a severe nitrogen-deposition in alpine meadows which often leads to phosphorus limitation of plant productivity. In these high-altitude localities, Cyperaceae have an increasing biomass while other functional groups decrease. Meanwhile, Cyperaceae are known to have the ability of producing dauciform roots, which are formed under phosphorus limitation, but in China, are only described in these high-altitude places. So, is the superiority of Cyperaceae and the formation of dauciform roots in high-altitude localities related to the accumulation of nitrogen? And is there a link between them? A *Carex filispica* dominated community in Baima Snow Mountain was selected and quantitative fertilization with four levels of nitrogen and three levels of phosphorus was performed. After 2 weeks, *Carex filispica* individuals with and without dauciform roots were separated and analyzed for their regular root properties, dauciform root properties, biomass and chemical traits of above- and belowground parts. The total cover of the community declined under phosphorus limitation with increasing nitrogen supply, while the relative cover difference of *Carex filispica* increased with increasing nitrogen supply and decreased with increasing phosphorus supply. Dauciform roots had a more significant response to nitrogen supply than to phosphorus supply and they were formed the most at a low supply of nitrogen. The biomass and root properties of individuals with dauciform roots were enhanced by nitrogen supply and inhibited by phosphorus supply, while those of individuals without dauciform roots were often enhanced by phosphorus supply. Individuals with and without dauciform roots showed two different mechanisms, and were limited by significantly different factors, which can explain the opposite performance of Cyperaceae after nitrogen and phosphorus supply in previous studies.

## Introduction

Phosphorus (P) is an essential element for the growth of plants, but most P in soil is not readily available for most plants, and plants can only absorb this by forming various root specializations with specific structures and physiology^1–3^. Among these plants with specialized roots, some Cyperaceae are known to produce dauciform roots (DR)^4^, which have a ‘carrot-like’ shape with a swollen axis and dense long root hairs, dissolving soil-sorbed P through root exudates^5^, and are generally considered an adaptation to low P availability^2^. The formation of DRs is enhanced by a low aboveground P concentration^6^, but is not related to aboveground nitrogen (N) concentration^7^. However, recent studies showed that their formation is also related to N supply^8^.

The growth of plants in grassland ecosystems at low altitudes is mainly limited by N^9,10^, while at higher altitudes, it is limited by P or both^11–13^: the mineralization rate of N declines with increasing altitude because of the low temperature, therefore more N can accumulate in the soil^14,15^, which intensifies the P limitation^13,16^. With increasing altitude, Cyperaceae, a dominant alpine meadow family, increase in biomass, while the role of Poaceae gradually decreases^17^. Also, in China, Cyperaceae with DRs were only found in alpine meadows in Yunnan Province^18^. This can be explained by that the accumulation of N and the limitation of P at high altitudes led to the formation of DRs, therefore, the advantage and dominance of Cyperaceae.

Indeed Cyperaceae are promoted by continuous N deposition^19^ and have the ability to adjust the traits for obtaining P in response to increasing N supply, which is considered one of the reasons why Cyperaceae often dominate after N fertilization in nutrient-limited environments^20^. Cyperaceae always has a unique performance in alpine meadows: a previous study found that N addition increased the height of Cyperaceae without any significant effects on Poaceae^21^, which can be explained by the fact that some Cyperaceae have the ability to produce DRs in response to the increasing N supply. On the other hand, however, there are also some studies found that N fertilization increased the cover and biomass of Poaceae, while having no effect on Cyperaceae^22–24^. There is still no definite conclusion about the response of Cyperaceae to N and P fertilization and the role of DRs, while we pose the following questions: (1) Is the dominance of Cyperaceae related to the extra N supply at high altitudes? (2) Does N accumulation enhance the growth of DRs while P accumulation has an inhibitory effect? (3) Can the presence of DRs affects the performances of Cyperaceae in alpine meadows? If so, do individuals with and without DRs have the same response to N and P supply?

The content of soil N and P can cause significant responses in functional traits of plants^25–27^, including variation in biomass, morphology and chemical traits, which can reflect the adaptation strategies of plants to environments^28^. Therefore, based on functional traits, this study investigated the short-term responses of DRs and other functional traits of *Carex filispica* to N and P fertilization under natural competition, analyzed the role DRs play under disturbed environment and the intra-specific difference between individuals with and without DRs, making an attempt to solve the above questions and providing a reference for future related studies.

## Methods

### Study area

The study is located at the Baima Xueshan Pass (99.08 E, 28.34 N) at 4320 m in Baima Xueshan National Nature Reserve, Diqing State, Yunnan Province, which is one of the few natural habitats in China where DRs were described^29^. The area is mainly an alpine shrub-meadow zone, with a cold-temperate mountain monsoon climate, an annual mean temperature of − 1.0 °C and precipitation of 600–650 mm^18^. The site is dominated by *Carex filispica* and *Polygonum viviparum*.

### Experimental design and measurement

Three replicate blocks were laid out across the site, each block was then subdivided into 12 squares (1 m × 1 m) for 12 treatments, in which four levels of N supply (as (CO(NH_2_)_2_) and three levels of P supply (as CaP_2_H_4_O_8_) were combined and performed. The N supply was 0, 10, 20, 40 g/m^2^, and the P supply was 0, 20, 50 g/m^2^ (hereafter described as ‘N0, N10, N20, N40, P0, P20 and P50’). Each block was at least 1 m from others to avoid interference, and the location of each treatment differed among blocks to avoid positional effects.

The first phase of the experiment had lasted for 2 weeks. 2 weeks after the treatments, the total community cover (TC) and absolute cover of *C. filispica* (ACC) were measured again, just like before the treatments. Each square was photographed and imported into Photoshop 2020 with a 10 × 10 intersection grid for calculation; each intersection of the grid with vegetation was recorded as a ‘hit’, and then multiplied by 100 to obtain the absolute cover values of both the community and of *C. filispica.* After the second photographing, 10 cm × 10 cm × 10 cm of plants and surrounding soil were excavated from each square, and all 602 individuals of *C. filispica* were separated from the soil, carefully washed and sorted by whether they had DRs. After which, 298 individuals with DRs were observed under a stereo-microscope, and the amount, size and color of DRs were recorded. The color of DRs was rated on a scale of 1–5 points, brightest to darkest. The formula of DR density is as follows: total amount of DRs/ the dry weight of roots. Using a LA-S root analyzer, the total length, surface area, volume and average diameter of the whole root system of individuals with and without DRs were measured respectively.

Following these measurements, the aboveground (leaves and fruits) and belowground (roots and rhizomes) biomass of individuals with and without DRs was oven-dried at 70 °C, weighed and grounded to measure the organic carbon (OC), total nitrogen (TN), and total phosphorus (TP) concentration. OC was determined by the potassium dichromate wet-oxidation method, TN was determined by the indophenol blue colorimetric method after digestion with H_2_SO_4_–H_2_O_2_, and TP was determined by the vanadium molybdate yellow colorimetric method after digestion with H_2_SO_4_–H_2_O_2_.

### Statistical analyses

To exclude the influence of other factors such as season, and to reflect the difference between *C. filispica* and the whole community, between each treatment and the control group, the absolute cover of *C. filispica* (ACC) was converted to relative cover (RCC = ACC/TC), then the total community cover difference (TCD), absolute cover difference (ACD) and relative cover difference (RCD) of *C. filispica* were calculated. The calculation were as follows:

$$\begin{aligned} {\text{TCD}} & = \left( {{\text{TC after treatment}}/{\text{TC before treatment}}} \right) \, \\ & \quad \times \, \left( {{\text{TC before treatment of control group}}/{\text{TC after treatment of control group}}} \right) \times {1}00 \\ \end{aligned}$$

$$\begin{aligned} {\text{ACD}} = & \left( {{\text{ACC after treatment}}/{\text{ACC before treatment}}} \right) \, \\ & \quad \times \, \left( {{\text{ACC before treatment of control group}}/{\text{ACC after treatment of control group}}} \right) \times {1}00 \\ \end{aligned}$$

$$\begin{aligned} {\text{RCD}} & = \left( {{\text{RCC after treatment}}/{\text{RCC before treatment}}} \right) \, \\ & \quad \times \, \left( {{\text{RCC before treatment of control group}}/{\text{RCC after treatment of control group}}} \right) \times {1}00 \\ \end{aligned}$$

All analyses were performed using SPSS Statistics (ver 19.0, IBM, Armonk, NY, USA), and figures were made using Origin (ver 2022, OriginLab, USA). Analysis of variance (ANOVA) were performed for the cover of *C. filispica,* biomass, DR properties, chemical traits and root properties to test the difference among the N and P fertilization. If there is any difference among them, LSD post-hoc tests were used to indicate the significant difference between two treatments. Statistical significance of the correlations among the treatments and chemical properties of *C. filispica* was tested by Pearson’s correlations, as well as cover and root properties.

## Results

### Cover

**Figure 1:**
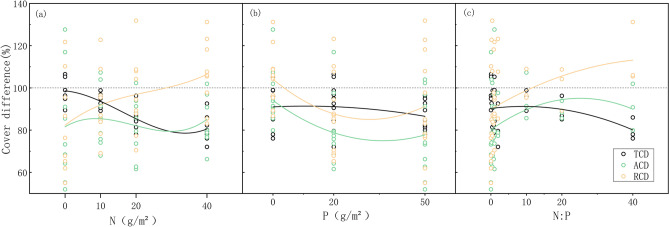
Variation of total community cover difference (TCD), absolute cover difference (ACD) and relative cover difference (RCD) of *C. filispica* with nitrogen (N) fertilization, phosphorus (P) fertilization and N-P fertilization ratio (N:P). The amount of N and P fertilizer applied to control group was recorded as 1 for the calculation of N:P fertilization ratio.

As can be seen in Fig. 1, TCD showed a significantly negative correlation with N fertilization (*r* = − 0.783, *p* = 0.000, Table 1) and showed a significant difference between the N0 treatments and both the N20 and N40 treatments for all P treatments (Table 2), while the RCD of *C. filispica* showed a tendency of positive correlation with N fertilization (*r* = 0.436, *p* = 0.053, Table 1). On the other hand, TCD was constant with the increasing P supply, while the ACD of *C. filispica* showed a negative correlation (*r* = − 0.377, *p* = 0.031, Table 1), especially when there was no extra N supply (Table 2).

**Table 1:** Analysis of Pearson correlation and variance result for the effects of nitrogen fertilization (N), phosphorus fertilization (P) and nitrogen-phosphorus fertilization ratio (N:P) on the total community coverage difference (TCD), absolute cover difference (ACD) and relative cover difference (RCD) of *C. filispica.*

	TCD		ACD		RCD	
	*r*	*p*	*r*	*p*	*r*	*p*
P	− 0.224	0.075	− 0.377*	0.031*	− 0.237	0.074
N	− 0.783*	0.000*	0.054	0.933	0.436*	0.053
N:P	− 0.290	0.001*	0.226	0.560	0.363*	0.086

*r* values represent the correlation coefficients; *p* values represent the levels of significance.

*indicates a significant difference or correlation (*p* < 0.05).

**Table 2:** Comparison of total community coverage difference (TCD), absolute cover difference (ACD) and relative cover difference (RCD) of *C. filispica* as dependent on nitrogen (N) and phosphorus (P) fertilization treatments.

	N0	N10	N20	N40
TCD				
P0	100 ± 2.8a	95 ± 2.9ab	89 ± 3.5bc	80 ± 3.0c
P20	102 ± 3.7a	95 ± 2.5ab	85 ± 1.1bc	81 ± 6.0c
P50	94 ± 2.1ab	91 ± 3.0b	81 ± 2.0c	80 ± 1.1c
ACD				
P0	101 ± 13.2a	95 ± 6.5ab	89 ± 2.4ab	91 ± 6.4ab
P20	83 ± 16.9ab	76 ± 4.6ab	72 ± 5.3b	85 ± 6.6ab
P50	60 ± 6.6b	86 ± 9.3ab	85 ± 11.8ab	80 ± 9.0ab
RCD				
P0	101 ± 10.5ab	100 ± 4.4ab	101 ± 5.9ab	114 ± 8.5a
P20	81 ± 14.6b	80 ± 5.6b	84 ± 7.2b	105 ± 11.0ab
P50	64 ± 6.3b	95 ± 13.8ab	106 ± 15.8ab	100 ± 10.2ab

Values represent the means of replicates ± SE (standard errors). Different letters indicate a significant difference among fertilization treatments (*p* < 0.05).

With increasing N fertilization and decreasing P fertilization, plant growth tends to be P-limited, which plants with dauciform roots are made to survive*.* TCD showed a decreasing trend with the increasing N:P ratio (Fig. 1c), which had a significant difference among the treatments (*p* = 0.001, Table 1), while the RCD of *C. filispica* showed a positive correlation and increased (*r* = 0.363, Table 1).

### Dauciform roots

**Figure 2:**
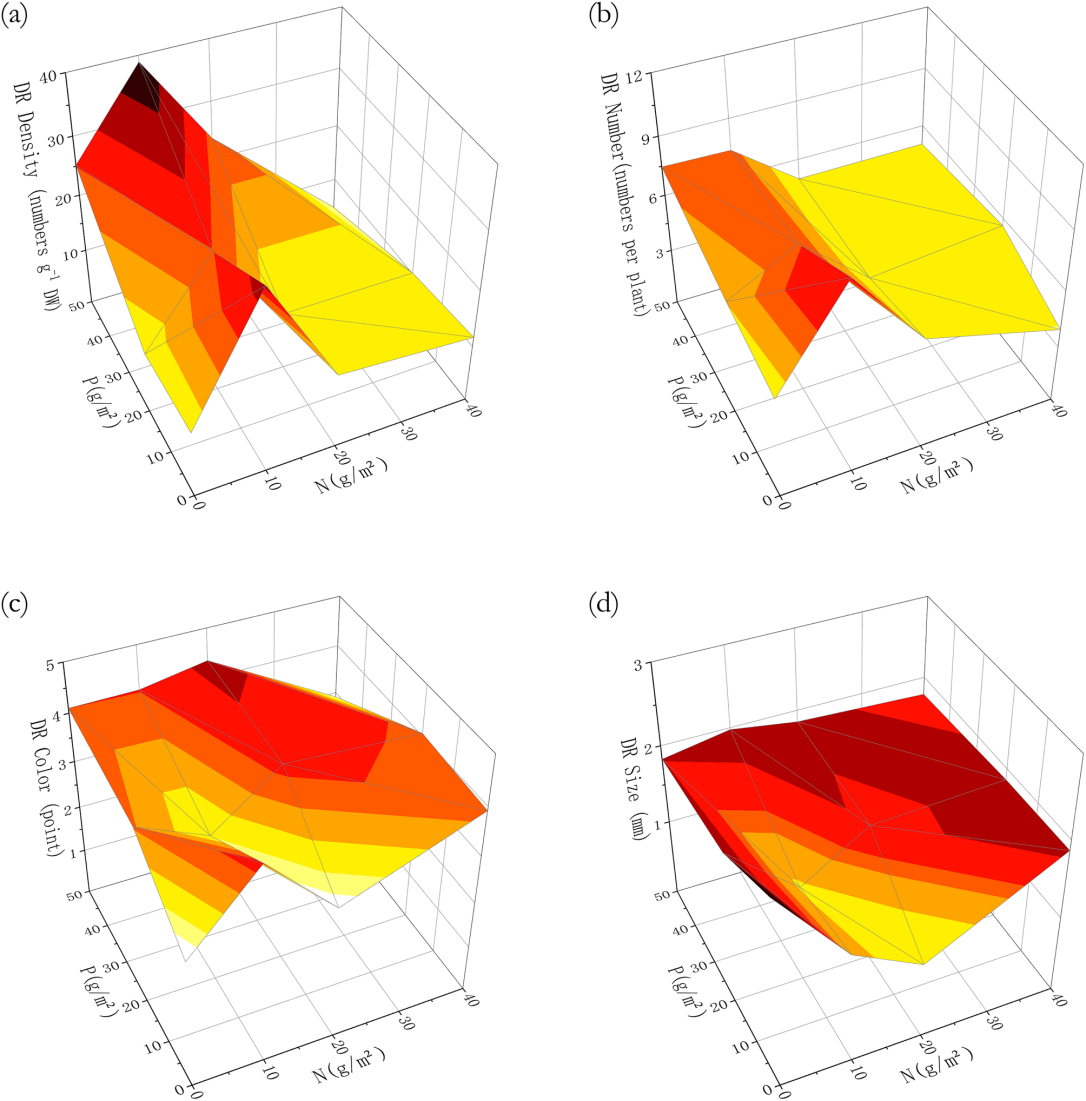
Differences among fertilization treatments in dauciform root (DR) properties: (**a**) DR density; (**b**) DR number; (**c**) DR color; (**d**) DR size.

All DR properties differed significantly with N fertilization treatment (*p* < 0.05, Table 3), except for the percentage of individuals with DRs (*p* > 0.05, Table 3), while P fertilization only had effects on the color of DRs. The density and number of DRs were at peak in N10 treatments (Fig. 2).

**Table 3:** Analysis of variance result for the effects of nitrogen (N) and phosphorus (P) fertilization on the percentage, density, number, size and color of DRs.

	Percentage		Density		Number		Size		Color	
	*F*	*p*	*F*	*p*	*F*	*p*	*F*	*p*	*F*	*p*
N	1.588	0.267	6.447	0.016*	7.371	0.000*	4.482	0.004*	6.579	0.000*
P	1.581	0.258	0.731	0.508	0.010	0.990	1.647	0.194	7.683	0.001*

*F* values represent F-ratio; *p* values represent the levels of significance.

*indicates a significant difference (*p* < 0.05).

### Biomass

**Figure 3:**
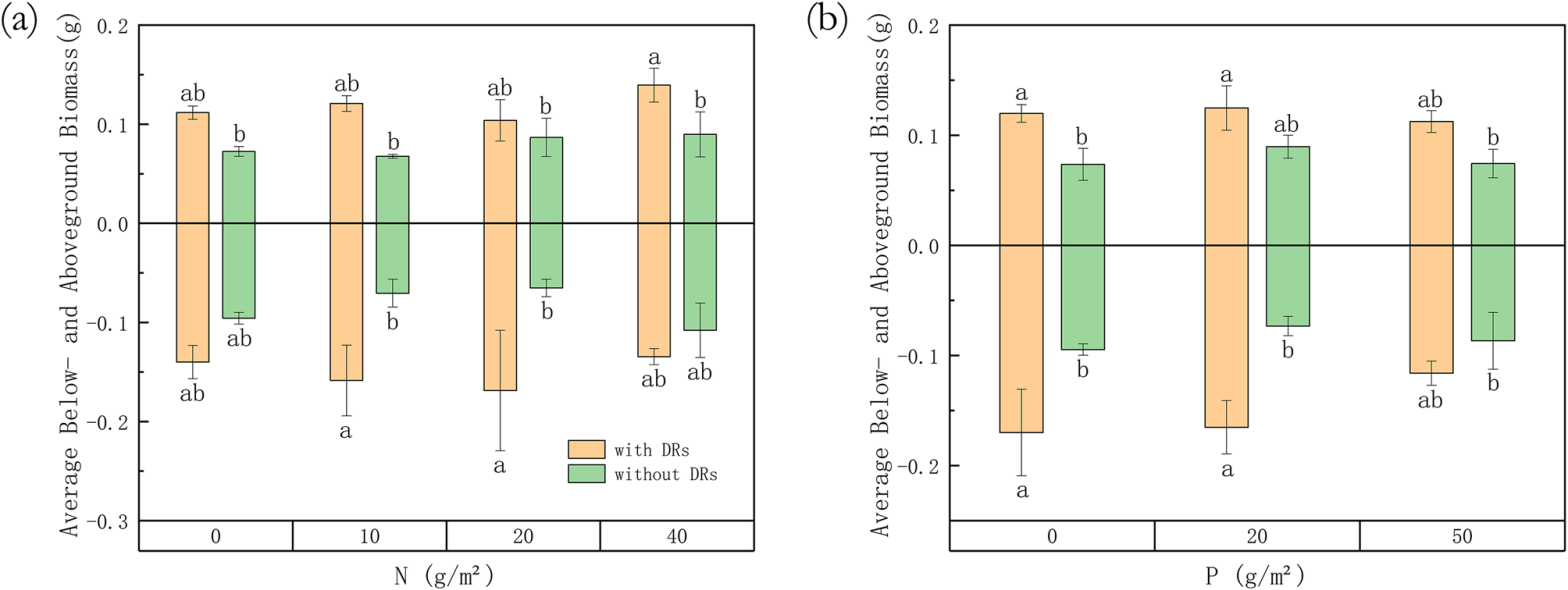
Differences between individuals with and without dauciform roots (DRs) as dependent on fertilization treatment in average above- and belowground biomass. Bars represent means ± SE (standard errors). Different letters indicate a significant difference among treatments (*p* < 0.05) separately for above- and belowground.

Both individuals with and without DRs showed no significant difference in their aboveground or belowground biomass as dependent on fertilization treatment, while individuals with DRs had both more average above- and belowground biomass, which often differed significantly from those without DRs (Fig. 3). Of these, the belowground biomass of individuals with and without DRs differed significantly in N10 and N20 treatments, while the aboveground biomass differed in N40 treatments (Fig. 3a). Individuals with and without DRs showed a significant difference in both their above- and belowground biomass when there was no extra P supply, while the aboveground difference vanished in P20 treatments and no significant difference left between them in P50 treatments (Fig. 3b).

**Figure 4:**
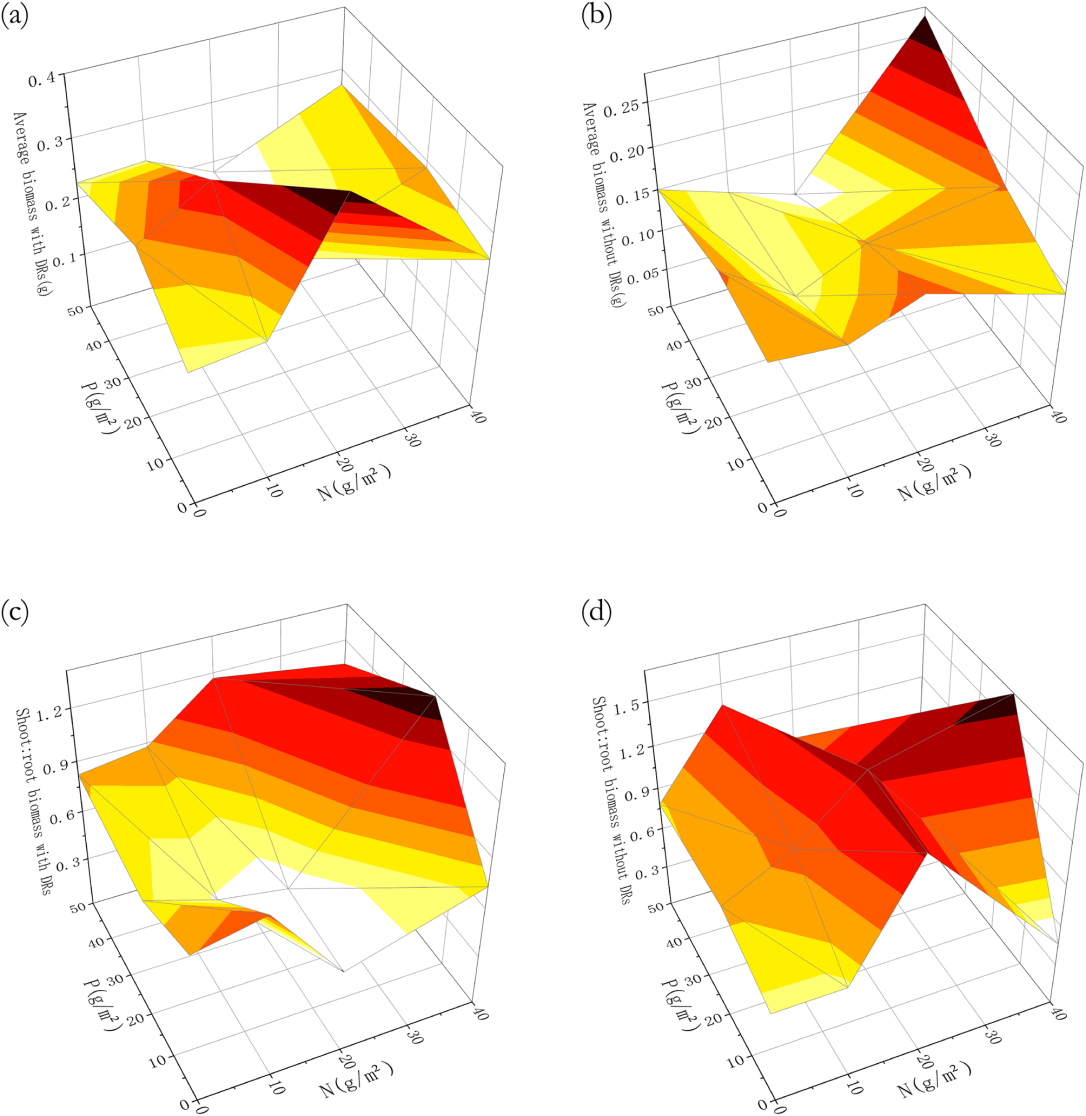
Differences among fertilization treatments in biomass: (**a**) average total biomass of individuals with dauciform roots (DRs); (**b**) average total biomass of individuals without DRs; (**c**) aboveground: belowground biomass ratio of individuals with DRs; (**d**) aboveground: belowground biomass ratio of individuals without DRs.

Similarly, Fig. 4 shows that the biomass of individuals without DRs reached the maximum after the highest phosphorus fertilization, while individuals with DRs were at maximum with no P fertilization. Individuals with and without DRs had a similar trend of aboveground: belowground biomass ratio, which was larger with abundant N and P, i.e. more biomass was put into aboveground.

### Chemical traits

**Figure 5:**
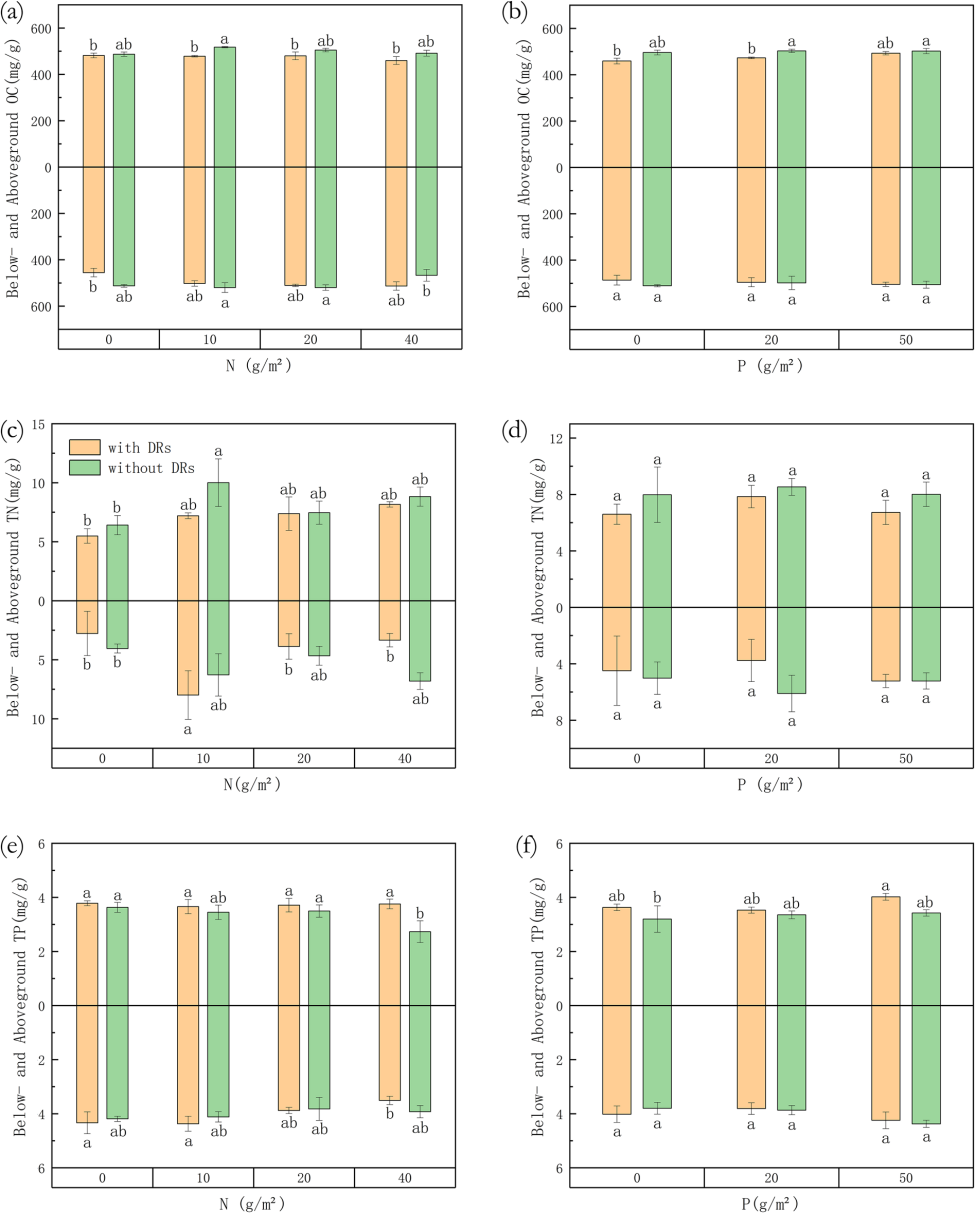
Differences among fertilization treatments in (**a**) below-/aboveground Organic carbon (OC) concentration with N fertilization; (**b**) below-/aboveground Organic carbon (OC) concentration with P fertilization; (**c**) below-/aboveground Total Nitrogen (TN) concentration with N fertilization; (**d**) below-/aboveground Total Nitrogen (TN) concentration with P fertilization; (**e**) below-/aboveground Total Phosphorus (TP) concentration with N fertilization; (**f**) below-/aboveground Total Phosphorus (TP) concentration with P fertilization. Bars represent means ± SE. Different letters indicate a significant difference among treatments (*p* < 0.05) separately for above- and belowground.

According to Fig. 5, in N10 treatments, individuals with DRs showed a significant increase in belowground N concentration whereas individuals without DRs showed a significant increase in aboveground N concentration in comparison with N0 treatment, both of which recovered after higher N fertilization (Fig. 5c). As mentioned before, the number of DRs peaked in N10 treatments, which may be correlated with the increase in the belowground N concentration. There was a significant difference in aboveground P between individuals with and without DRs at the highest concentration of N supply (Fig. 5e). What is noteworthy is that there was no significant difference in phosphorus or nitrogen concentration after different P fertilization (Fig. 5d and f).

**Figure 6:**
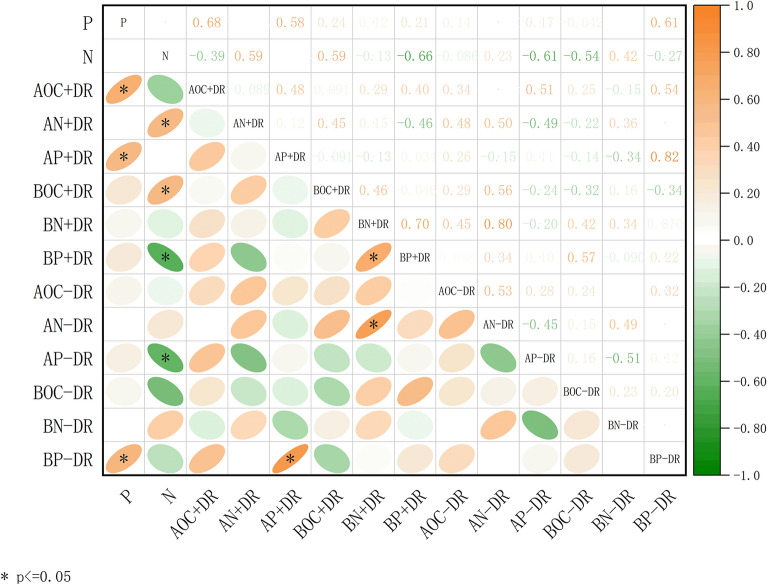
Relationships among fertilization treatments and chemical traits of *Carex filispica*, described by Pearson correlation coefficients, separated by Dauciform root (DR) presence. AOC, aboveground organic carbon; AN, aboveground nitrogen; AP, aboveground phosphorus; BOC, belowground organic carbon; BN, belowground nitrogen; BP, belowground phosphorus; + DR, individuals with dauciform roots; − DR, individuals without dauciform roots. * indicates a significant correlation (*p* < 0.05).

**Figure 7:**
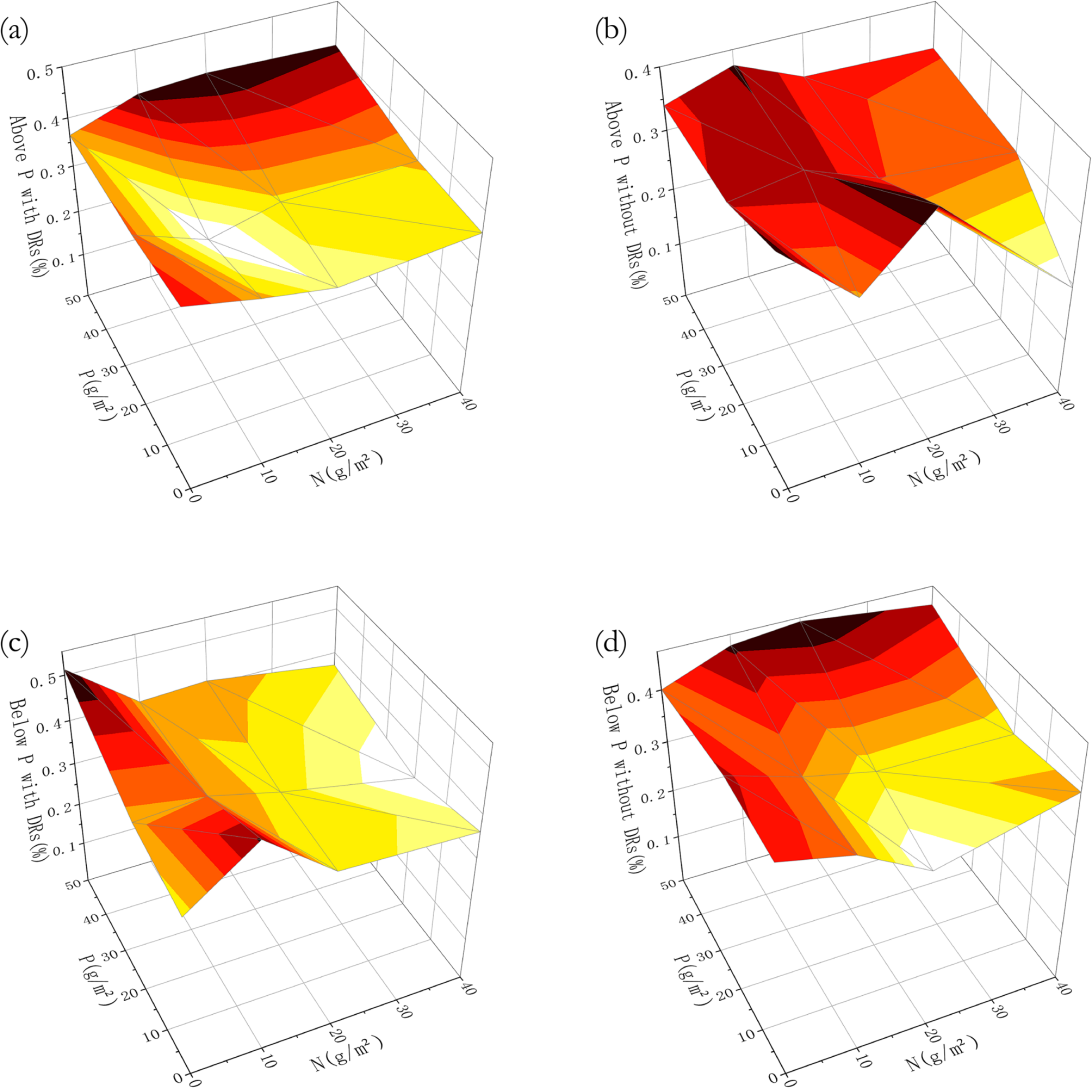
Differences among fertilization treatment in phosphorus (P) concentration: (**a**) aboveground P concentration of individuals with DRs; (**b**) aboveground P concentration of individuals without DRs; (**c**) belowground P concentration of individuals with DRs; (**b**) belowground P concentration of individuals without DRs.

P fertilization was positively related to the aboveground P of individuals with DRs and the belowground P of individuals without DRs, while N fertilization was negatively related to the belowground P of individuals with DRs and the aboveground P of individuals without DRs (*p* < 0.05, Figs. 6 and 7), which showed a significant inter-specific difference: for individuals without DRs, the aboveground P was associated with N fertilization and showed P-deficiency with increasing N fertilization, while individuals with DRs were not affected by N and increased with P concentration.

### Root properties

The effect of N or P fertilization on root traits was not significant, while there were significant differences in the length and surface area between individuals with and without DRs (*p* < 0.05, Table 4). As can be seen in Table 4, the root length and surface area showed a positive correlation with DR presence (*r* = 0.308), which means that those properties of individuals with DRs were significantly higher than those without.

**Table 4:** Analysis of Pearson correlation and variance result for the effects of nitrogen, phosphorus and dauciform root (DR) presence on the length, surface area, volume and diameter of nondauciform roots.

	Length		Surface area		Volume		Diameter	
	*r*	*p*	*r*	*p*	*r*	*p*	*r*	*p*
P	− 0.152	0.051	− 0.142	0.148	− 0.102	0.656	0.006	0.679
N	0.177	0.456	0.127	0.573	0.045	0.658	− 0.111	0.240
DR	0.308*	0.007*	0.236*	0.040*	0.137	0.258	− 0.049	0.681

*r* values represent the correlation coefficients; *p* values represent the levels of significance.

*indicates a significant difference (*p* < 0.05).

**Figure 8:**
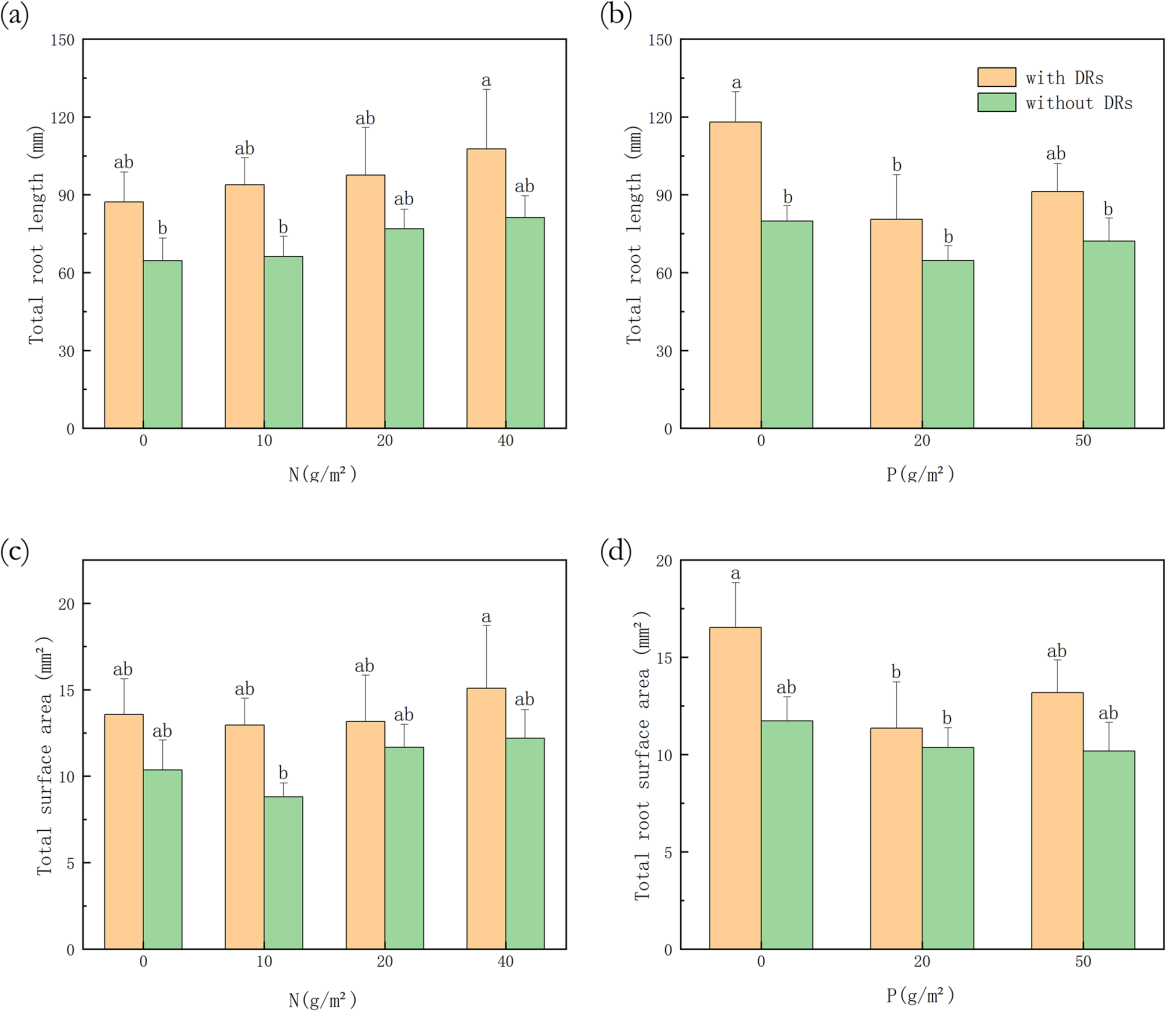
Differences among fertilization treatments in (**a**) total root length with nitrogen (N) fertilization; (**b**) total root length with phosphorus (P) fertilization; (**c**) total root surface area with N fertilization; (**d**) total root surface area with P fertilization. Bars represent means ± SE. Different letters indicate a significant difference among treatments (*p* < 0.05).

**Figure 9:**
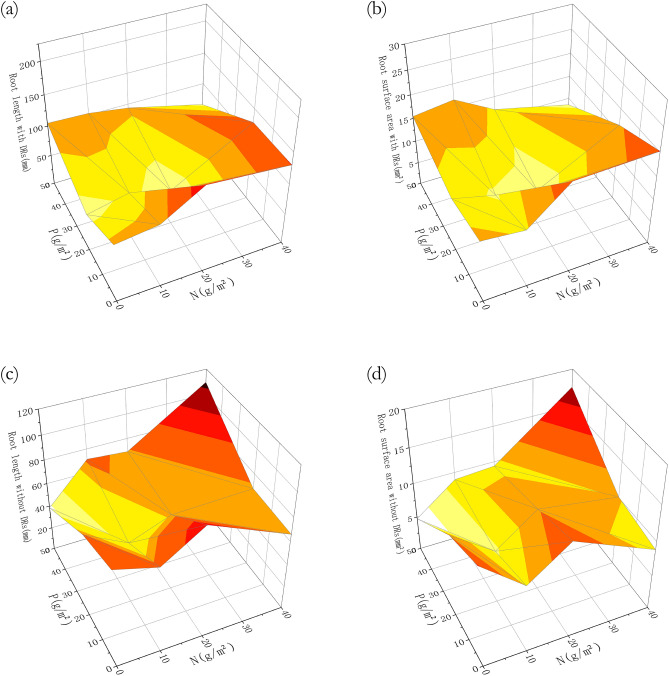
Differences among fertilization treatments in root properties: (**a**) total root length of individuals with DRs; (**b**) total root length of individuals without DRs; (**c**) total root surface area of individuals with DRs; (**b**) total root surface area of individuals without DRs.

As can be seen in Fig. 8, without P fertilization, there was a significant difference in root length between individuals with and without DRs (Fig. 8b). Furthermore, the root length and surface area of individuals with DRs was greater and both showed a significant decrease in P20 treatments, comparing to P0 (Fig. 8b and d). Not unlike the biomass variation (Fig. 4), the root length and surface area of individuals with DRs was also at maximum with no P and high N fertilization, while individuals without DRs reached the maximum after both the highest N and P fertilization (Fig. 9), which reflected the limiting factor partially.

## Discussion

### The relative cover of* C. filispica* was inhibited by P fertilization and promoted by N fertilization, unlike the cover of community

Currently, there is no definite conclusion in the response of Cyperaceae to N and P fertilization. N supplementation significantly increases the inorganic and total N content in the soil while decreasing the available and total P content, which relieves the N-limitation but may cause P-limitation and inhibit the growth of the community^30,31^. After N fertilization, the competition for available P becomes crucial, which may encourage the formation of traits that favor this competition^32^. In this study, the total cover of the community decreased with increasing N fertilization, while the RCD of *C. filispica* increased with N fertilization and decreased with P fertilization. This result is consistent with previous researches that Cyperaceae has a strong adaptation to P-limitation and that its growth improves with N-fertilization^21^. This is possibly caused by the production of DRs that provides Cyperaceae with an advantage in the competition for P^6^.

Meanwhile, there is also research showing that N application significantly increases the cover of Poaceae, while having no effect on Cyperaceae^22^. On the one hand, these conflicting phenomena may be related to both the concentration of natural soil fertility and N fertilization: mild N addition adds nutrients to the soil and promotes the growth of Poaceae which has no N-fixing ability themselves^23^, while higher N addition leads to P-limitation and provides Cyperaceae with an advantage. On the other hand, this may also be related to whether Cyperaceae in these studies could or did produce DRs, given that our study found that plants with and without DRs responded differently to N and P fertilization in several ways, as we will explain further.

### N fertilization had a more significant effect on dauciform roots of *C. filispica*, than P fertilization did

The screening of functional traits is not only between species but also within species^33^: species and individuals without the ‘suitable’ traits tend to be eliminated by the environments. While the abundant species tend to be those with greater competitiveness for the limited resources^34^, the very theory can also be applied to the intra-specific selection. DR density was higher at both ends of the P-fertilization (P0 and P50) (Fig. 2a), indicating that individuals with DRs were more competitive at these times, which is similar to and may share the same explanation with the study of cluster roots: under P-limitation, they are invested to assist with P-acquisition, while with abundant nutrients, plants have too much nutrients to save the costs therefore they can be produced as well^35^ (Fig. 2a).

There are many previous studies that have shown that P-concentration has a significant effect on the growth of both DRs and Cyperaceae^2,6,36^. A Japanese study found that the number of DRs was negatively correlated with aboveground P-concentration but not with aboveground N-concentration^7^. However, several results from this study suggested that the effect of N fertilization was more significant than P. In our study, the number of DRs was at peak in N10 treatments, which was consistent with a previous study from Australia: The number of DRs increased significantly and then decreased with the increasing N supply^4^.

Besides, individuals with DRs did not show any significant difference in N or P concentration with different P-fertilization. In the meanwhile, belowground N was also at peak in N10 treatments, as well as the number of DRs. Proper N addition increases community production, while an over-application imbalances the nutrient of soil and plants^37,38^, causes soil acidification and toxic effects on plants^39,40^, which harms community productivity^9^. Meanwhile, P supply appears to have no significant effect, which can be explained by the fact that the leaf P showed no evidence of P deficiency in all treatments.

### Individuals without DRs tend to be promoted by P fertilization, while individuals with DRs tend to be promoted by N fertilization: they might be two groups limited by different factors with different mechanisms, which can be responsible for the opposite performance of Cyperaceae after N and P fertilization

The "functional equilibrium" hypothesis suggests that plants tend to allocate more biomass to organs absorbing the most limited resources^41,42^, with leaves for light, roots for nutrients and water^43^. This relationship, however, mostly applies to solo growing plants, which becomes more challenging and complex when the neighboring individuals are competing for the same resources^44,45^. Individuals with and without DRs differed significantly in biomass changes: the biomass of individuals with DRs was generally higher than those without, P-fertilization costed individuals with DRs their biomass advantage, while N-fertilization widened the gap between these two groups. After low and moderate N-fertilization (N10 and N20), individuals with DRs had a significantly higher belowground biomass than without (Fig. 3), while the number of DRs and belowground N concentration also had a significant increase (Figs. 2 and 5c), which may be explained by the increase of DRs which may have caused more inputs to the belowground parts. Even though the trends in biomass were different, the aboveground: belowground biomass ratios of individuals with and without DRs showed a consistent trend, with the leaf biomass ratio increasing with nutrients, indicating that plants invest energy in aboveground light competition when belowground nutrients are sufficient^42^.

Similar to the changes in biomass, root length and surface area of individuals with DRs showed a tendency of increasing with N fertilization and showed a significant decrease in P20 treatments in comparison with no P fertilization, while individuals without DRs showed no such difference (Fig. 8). As the degradation intensified, the total root length and surface area showed an increasing trend, while volume tended to be stable, indicating that the root system increased its absorbing ability by decreasing diameter and increasing surface area^46^, which helps to adapt to the extreme environment^47^. In our study, individuals with DRs showed the same tendency as above: they produced a large amount of fine roots, perhaps DRs, under N-abundant P-limitation situation to help with P-absorption, so that they can engage in aboveground competition. A theory suggested that the plasticity of belowground morphological traits may be genetically limited^48^, therefore are not as important as aboveground morphological traits with regards to resource acquisition^49^. In our study, there was a significant difference in root length surface area between individuals with and without DRs, while N and P fertilization had no effect, which can be explained by the above theory.

Changes in plant physiology and morphology can be caused by external factors, which may become a maintenance cost once the stimulus is removed, while physiology is faster and more efficient than morphological plasticity response^42^^50^. Morphological traits tend to be more conservative and stable^51^, and as we mentioned before, root morphology did not differ significantly after N and P fertilization, whereas chemical traits are highly unstable and more sensitive to nutrients^52^. Güsewell's experiment showed that aboveground phosphorus concentrations in Cyperaceae decreased with increasing N application and increased with increasing P application^8^, while in this study, individuals with and without DRs were discussed separately: The aboveground P concentration of individuals with DRs increased with P fertilization, while their belowground P decreased with increasing N fertilization, this is because the soil became P-limited with increasing N fertilization, while individuals with DRs produced DRs that helped to absorb nutrients from the P-limited soil and invest energy into aboveground competition, therefore maintain aboveground P concentration, suggesting that the presence of DRs can protect aboveground growth from P-limitation caused by excessive N fertilization. What is noteworthy is that individuals without DRs have the exact opposite trend of aboveground and belowground P changes, with their belowground P increasing with P fertilization and aboveground P limited by N fertilization. Previous study showed that comparing to shoot, the P concentration of root can be more reflective of soil available P^53^, which only seems to apply to individuals without DRs.

The filtering function of functional traits in plants is not only among species but also within species^33^, intra-specific variation in functional traits should also be considered in the understanding of community mechanisms^54^. Whether plants with and without DRs should be divided into two functional groups has caused heated debate^55^, while this study found that individuals with and without DRs differed significantly in biomass, chemical traits, and root morphology, on top of that, one is promoted by N fertilization and the other by P fertilization. Therefore, they might be two groups limited by different factors with different mechanisms that can be discussed separately in future studies. Meanwhile, this difference in the mechanism can be responsible for the opposite performance of Cyperaceae after N and P fertilization in alpine meadows.

## Conclusion

This study investigated the differences in functional traits by separating individuals of *C. filispica* with and without DRs in an experiment with quantitative application of N and P fertilization, concluding that (1) the cover of *C. filispica* showed an advantage with the increasing N and decreasing P, therefore their dominance at high altitudes could be related to the extra N supply; (2) DRs showed a more significant response to N supply than to P supply and they were formed the most at a low supply of N; (3) individuals with DRs showed an increase in biomass and root properties with N supply and a decrease with P supply, while those traits of individuals without DRs were often enhanced by P supply. Individuals with and without DRs differed in various dimensions and had significant intra-specific differences in response to N and P fertilization. However, this study was limited to the response 2 weeks after the fertilization, which can not reflect the long-term response, therefore further studies are still needed.

## Data Availability

Data are available on request to the corresponding author.
